# Improving Nanoparticle Size Estimation from Scanning Transmission Electron Micrographs with a Multislice Surrogate Model

**DOI:** 10.1021/acs.nanolett.4c06025

**Published:** 2025-01-29

**Authors:** Henrik Eliasson, Rolf Erni

**Affiliations:** † Electron Microscopy Center, Empa − Swiss Federal Laboratories for Materials Science and Technology, Überlandstrasse 129, 8600 Dübendorf, Switzerland; ‡ Department of Materials, ETH Zürich, CH-8093 Zürich, Switzerland

**Keywords:** Nanoparticles, Scanning transmission electron microscopy, Characterization, Machine learning, Synthetic data, Heterogeneous catalysis

## Abstract

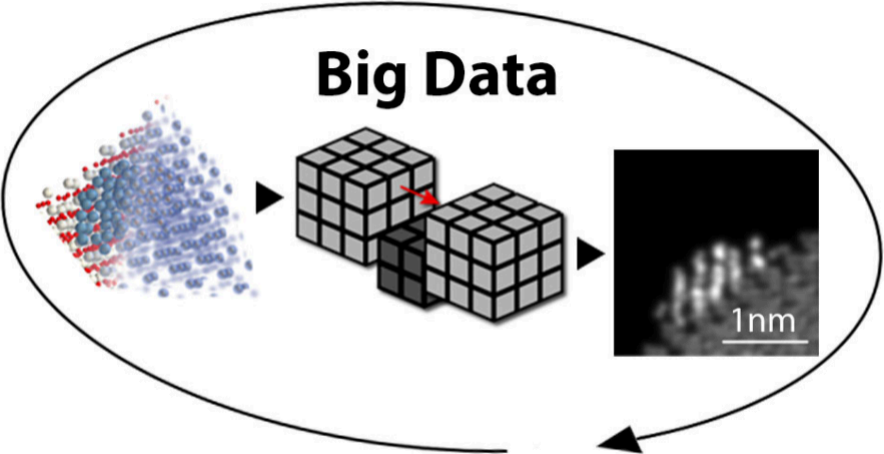

The computational cost of simulating scanning transmission electron microscopy (STEM) images limits the curation of large enough data sets to train accurate and robust machine learning networks for deep feature extraction from atomically resolved STEM images. For nanoparticle size estimation in particular, a diverse data set is essential due to the large variations in size, shape, crystallinity, orientation, and dynamical diffraction effects in experimental data. To address this, we train a 3D convolutional neural network to predict STEM images from voxelized atomic models, achieving a 100x speed-up compared to traditional multislice simulations while maintaining high image quality. We then generate a data set of 100.000 synthetic multislice images and investigate the performance of different size-estimator architectures as a function of training set size. A ResNet18-based model trained on 4000 real and 100.000 synthetic images is found to perform the best, reducing the median size-estimation error from 9.89% without synthetic data to 5.26%.

Precise and automatable atomic scale characterization techniques are needed to establish synthesis–structure–property relations in heterogeneous catalysis.^1,2^ High-angle annular dark-field scanning transmission electron microscopy (HAADF-STEM) has in the past decade cemented itself as the go-to tool for direct visualization of surface-supported catalyst species on the atomic scale, but current data-analysis workflows either lack precision,^3^ are specialized for single atoms catalysts,^4,5^ or are not unifiable with experiment automation due to particle orientation requirements.^6,7^ Recently, we explored a general approach to nanoparticle size-estimation that requires no human input and promises accurate size predictions for particles in a large size span and imaged in any orientation.^8^ This technique utilizes a CycleGAN^9^ to bridge the gap between experimental images of nanoparticles and physical multislice image simulations, allowing the generation of synthetic experimental data with known atomic model ground truths that can be used to train neural networks for nanoparticle size estimation in a supervised manner. The technique was applied to platinum particles supported on ceria (Pt/CeO_2_), which is a versatile catalytic system. Although performance was good, the study was limited by the size of the multislice data set and consequently the upper bound of this promising technique is still unclear.

Generating accurate multislice images of medium sized atomic structures (<500 nm^3^) is costly and can take anywhere from minutes to hours,^10^ even with GPU acceleration. Curating a large data set of tens of thousands of images, which is common for other specialized computer vision tasks,^11,12^ is thus unfeasible without access to several GPU nodes at HPC facilities or by trading accuracy for speed with more optimized algorithms like PRISM.^13,14^ An effective alternative to generating more physical image simulations is to leverage neural networks or handcrafted algorithms to generate synthetic data.^15−18^ However, for the task of nanoparticle size estimation from HAADF-STEM images, significant information is encoded in the pixel intensities due to the mass–thickness contrast of the modality,^19^ and it is likely that the synthetic data must be close to physical in order to be useful.

We approach this challenge by developing a surrogate model for multislice image simulations and train it on the data set presented in our previous work^8^ of paired atomic models and multislice HAADF-STEM images of Pt nanoparticles supported on CeO_2_ (Figure 1). The model architecture is chosen to be a 3D U-Net which operates on volumetric data, allowing the model to learn depth relations in contrast to other works where a simple projected image of the atomic model acts as the starting point.^20^ We then use the surrogate model to generate a large data set of 100.000 synthetic multislice images of Pt particles on ceria and explore the performance of different size-estimator architectures (the U-Net used in our previous work,^8^ ResNet18, and ResNet50^21^) as a function of number of synthetic images in the training set. In the end, the optimized models are applied to real experimental images of Pt particles on ceria, and their performance is qualitatively evaluated.

**Figure 1:**
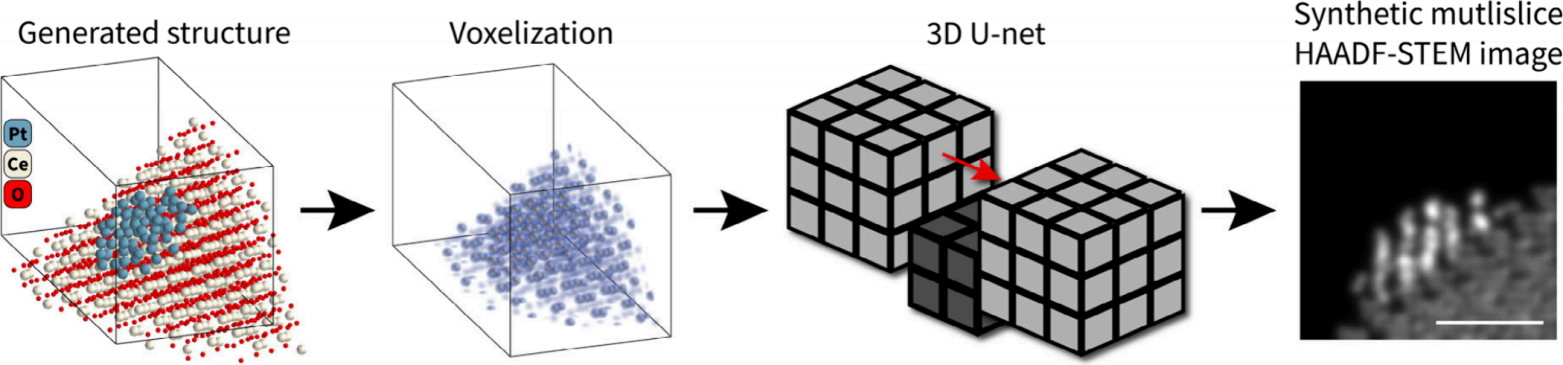
An atomic model is voxelized into a 128 × 128 × 256 volume and sent through a 3D U-Net to predict the multislice HAADF image of the original atomic model. The scale bar is 1 nm.

Predicting an image from an atomic model involves translating between two different modalities, from point cloud to image. While powerful techniques such as PointNet effectively process point clouds directly,^22^ their implementation remains complex and would require extensive customization to output images along a specific optical axis through the point cloud. Instead we chose to voxelize the atomic models into 3D volumes and utilize a 3D U-net^23^ to predict the 2D image from the input. This approach leverages the inherent spatial biases of convolutional networks, facilitating more straightforward predictions of 2D images from the 3D input volumes at the cost of negligible interpolation errors introduced by the voxelization. The atomic models are voxelized to a 256 × 128 × 128 volume by placing Gaussian balls at each atom position with an amplitude equal to *Z*/118 and a standard deviation of , where *Z* is the atomic number of the atom and *d* is the diameter of the electron beam which we have set to 0.5 Å. Each Gaussian ball is cut off at 3σ, and the complete model is finally shifted such that the first nonzero voxel in the depth is placed at z = 0.

The 3D U-net (implementation available on GitHub^24^) is adapted to output a 2D image by adding a 3D convolution in the final layer that collapses the depth dimension but otherwise maintains a standard architecture with 3 downsampling steps, 3 upsampling steps, and skip connections between corresponding layers in the encoder and decoder. We train the model on 4000 out of the 5000 image–model pairs from the data set and use the remaining 1000 for validation. The model was trained for 100 epochs with a batch size of 2 using the Adam optimizer and a cosine annealing learning rate scheduler from 1e-4 to 1e-6. As loss function we use mean squared error (MSE) for the first epoch and a sum of MSE scaled by 10^4^ and a perceptual LPIPS loss calculated with squeeze net^25,26^ for the remaining epochs. The perceptual loss is used because it helps to retain detail and avoid oversmoothening in the generated images. The large scale factor for the MSE loss is needed to bring the two loss terms to the same scale, as the pixel intensities of the images are very small.

Generating an atomic model (∼5 s), creating its voxel representation (∼0.66 s), and generating an image from it (∼0.08 s) takes about 6 s on our workstation equipped with an RTX 4090 GPU. That corresponds to a speed-up of about 2 orders of magnitude compared to the average simulation time of about 10 min using the GPU accelerated multislice algorithm in Dr. Probe.^27^ However, it should be noted that the bulk of the computation time with the machine learning approach comes from generating the structures themselves, which must also be done for the multislice approach, and the speed-up is closer to 1000× if we only compare with voxelization and image generation (inference). Full details about the multislice data set and simulation settings can be found in our previous work.^8^ In short, the images are 128 × 128 with pixel sizes ranging from 5 to 35 pm, the cells are 7 × 7 × 7 nm or smaller, the slice thickness was 0.1 nm, and we use 10 frozen lattice configurations.

In general, the generated images are highly detailed and could easily be mistaken for real multislice images (Figure 2). To quantitatively evaluate the model we use LPIPS as a perceptual metric and 1 – R^2^, where R^2^ is the coefficient of determination, as in the work of Combs.^20^ Our model achieves an average LPIPS of 0.00293 and an average 1 – R^2^ of 0.00710 on the 1000 images in the validation set. Looking closely, there are typically some slight intensity differences where the model was not able to accurately model the dynamical diffraction of the electrons in the sample, which presents itself as deviations from the true value by about 0–20%. This effect is most pronounced in images of samples aligned along a major zone axis where channeling effects are present,^28,29^ as seen in the fourth column of Figure 2. We believe this struggle to handle both images in and out of the zone axis could be aided by more training data but also by playing around with the fraction of training images of structures in the zone axis (currently about 20–40% of the set). Finally, we observe that the model handles images with different pixel sizes well.

**Figure 2:**
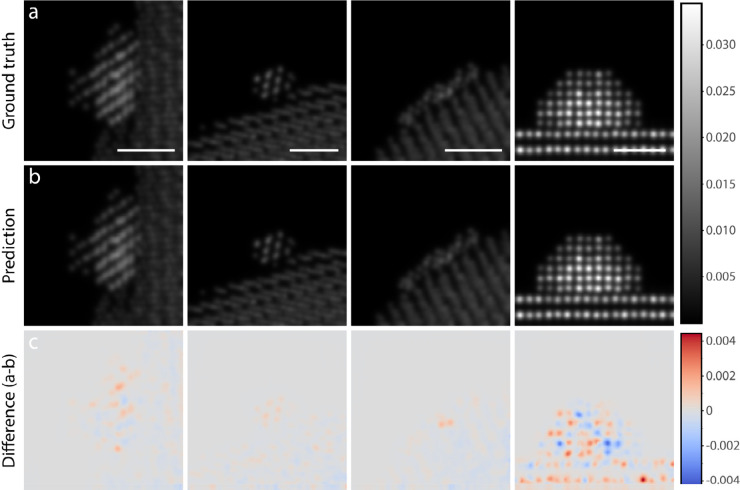
(a) Selected ground truth HAADF-STEM multislice image simulations. Scale bars are 1 nm. (b) Corresponding synthetic multislice images generated by the 3D U-Net. (c) The pixel-wise difference between ground truth and prediction calculated by subtracting the prediction from the ground truth. The difference can be compared directly to the pixel intensity of the images in rows a and b.

Utilizing the trained multislice surrogate model and the particle structure generator from our previous work,^8^ a large data set of 100.000 synthetic multislice images of Pt particles on CeO_2_ was generated. The data set contains particles in the range 1–1000 atoms, and about 40% of the images have either support or particle or both aligned to a major zone axis (the same distribution as in the real multislice set). The idea is now to use this synthetic data set along with the real multislice data set of 5000 to train a size-estimator network to output the size of an imaged nanoparticle in number of atoms. Furthermore, we are only concerned about the performance of the size-estimator on real experimental data; thus, we use the trained CycleGAN from our previous work to map the synthetic and real multislice sets into the noisy experimental domain (Figure 3a). We refer the reader to our previous work for details about the experimental STEM data.^8^ Now we have 105.000 synthetic experimental images with paired atomic models, out of which 5000 are based on real multislice images and 100.000 are based on synthetic multislice images. Out of the 5000 based on real multislice images, 1000 are used for validation, and the remaining 4000 are part of the training set. Unless explicitly stated otherwise, all mentions of multislice images henceforth concern multislice images mapped into the noisy experimental domain, and the prefix “real” or “synthetic” indicates if the images are mapped from real or synthetic multislice images.

**Figure 3:**
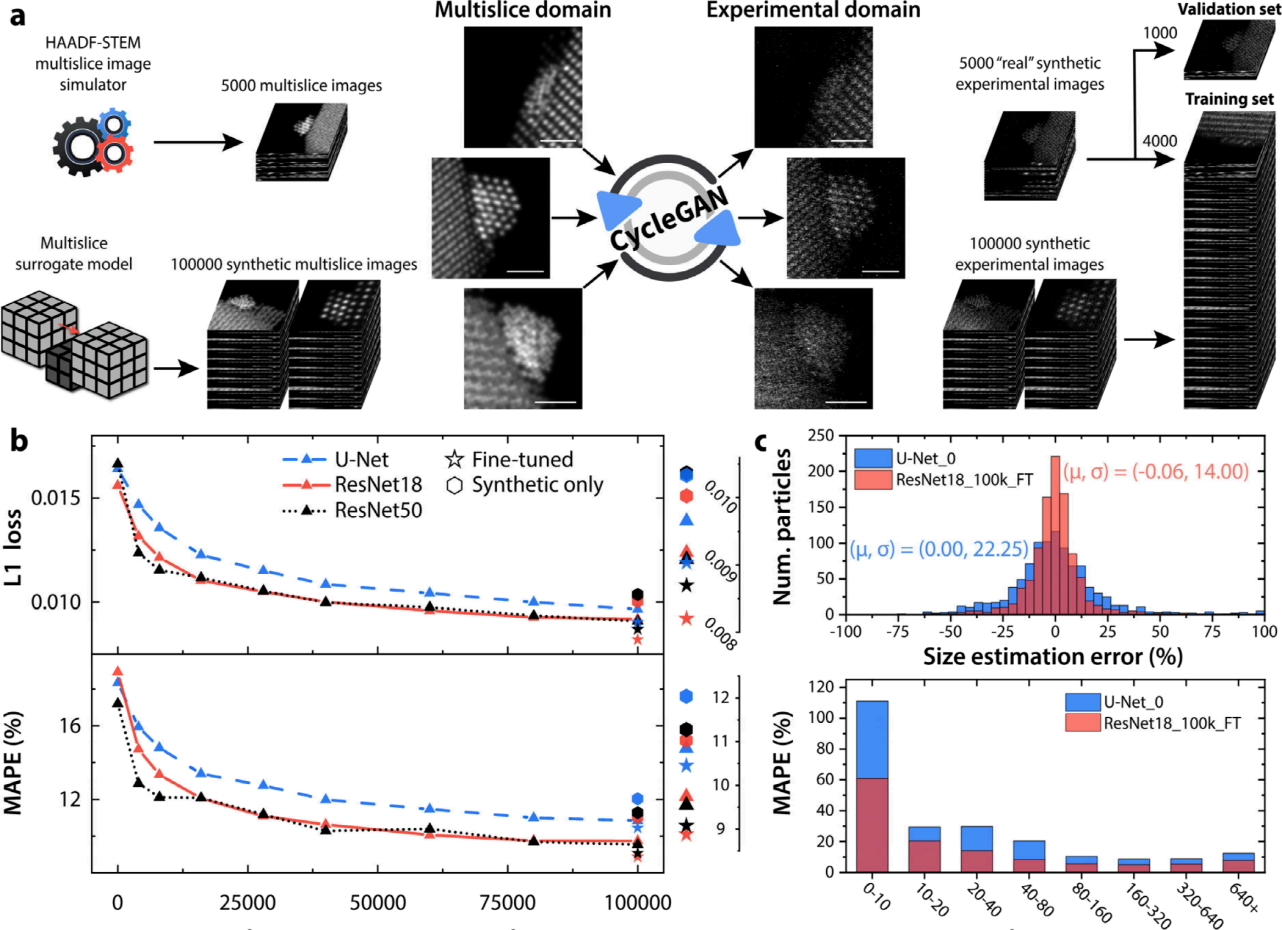
(a) Flowchart of the data set generation, real and synthetic multislice images are mapped to the experimental domain by sending them through a cycleGAN. Scale bars are 1 nm. (b) L1 loss and MAPE of U-Net, ResNet18, and ResNet50 as a function of number of synthetic images in the training set. (c) Size estimation error of all particles in the validation set for selected models and the particle size-dependence of the MAPE.

We compare three different network architectures for the size-estimator: the U-Net used in our previous work (17 M parameters), and ResNet18 (11 M parameters) and ResNet50 (24 M parameters), two established, size-efficient models widely used in various computer vision tasks.^21^ To explore the effectiveness of adding synthetic data, we train each model with 4000 real multislice images plus *x* synthetic ones, where *x* is selected from X = {0, 4000, 8000, 16000, 28000, 40000, 60000, 80000, 100000}. Each model configuration is trained five times with random parameter initialization and a different random selection of *x* synthetic images. The models are trained for 300 epochs using the Adam optimizer, an L1 loss function, cosine annealing with an initial learning rate of 0.001 and a 5 epoch linear warmup (selected after a brief parameter search), and a batch size of 128. The exact model implementations and links to the data sets can be found on GitHub.^24^ Both the L1 loss and the mean absolute percentage error (MAPE) are tracked each run. The L1 loss punishes errors on larger particles more heavily, while the MAPE punishes errors on small clusters more heavily. Tracking both metrics gives a good feeling for how well the model handles particles in different parts of the size spectrum (Figure 3b).

All models perform better with more synthetic images, and each architecture performs the best when trained on the complete synthetic set. The U-Net lags behind the ResNet variants, and we find ResNet18 to be more stable than ResNet50, likely due to the significantly larger size of ResNet50. ResNet50 would probably overtake ResNet18 with a more specialized training recipe and a larger data set. The models were also trained without any real multislice images which led to a decrease in performance (Figure 3b), but still significantly better than the models trained without synthetic data, highlighting the quality of the synthetic data. Furthermore, the best weights of each model were fine-tuned on the 4000 real multislice images for 100 epochs using cosine annealing and a 5 epoch linear warmup. The initial learning rates were set to 0.0001 for both ResNet18 and ResNet50 and 0.001 for the U-Net. Comparing the models trained without synthetic data with the fine-tuned ones, the L1 validation loss drops from 0.0156, 0.0166, 0.0164, to 0.0082, 0.0087, and 0.0090, for ResNet18, ResNet50, and the U-Net, respectively. Similarly, the MAPE drops from 18.934%, 17.205%, and 18.334% to 8.882%, 9.078%, and 10.452%. The fine-tuned ResNet18 model (ResNet18_100k_FT) achieves both the lowest L1 loss and the lowest MAPE on the validation set. To contextualize the improvement of ResNet18_100k_FT compared to our previous work with a U-Net trained with no synthetic data (U-Net_0), we take a deeper look at the particle size predictions on the 1000 validation images (Figure 3c). ResNet18_100k_FT reduces the median absolute percentage error (MdAPE) of size predictions from 10.64% for U-Net_0 to 5.26% (MdAPE for ResNet18_0 is 9.89%). After pruning a handful of predictions with a larger than 100% error (4 for ResNet18 and 18 for U-Net), we find that the error standard deviation is also significantly reduced from 22.25% to 14.00% with ResNet18_100k_FT. Furthermore, as the prediction error is sensitive to the particle size, the particles are divided into 8 size ranges and the MAPE was calculated for each interval (see Figure 3c). ResNet18_100k_FT increases the accuracy across all particle sizes with a particularly significant improvement for particles in the 0–10 atom size range, where the error is reduced from 111% to 61%. This still relatively large number for the smallest size interval can be explained by the L1 loss function used for training and the noise level of the data. If highly accurate predictions for a specific size range are desired, one would be better off training a model for that specific size range instead of a general one for sizes from 1 to 1000 atoms as presented herein.

In a final experiment to verify that the effort and computation time invested into generating synthetic data are worthwhile compared to simply generating more real multislice images, we generate 1000 more multislice images. The computation time of 1000 multislice images is comparable to generating 100000 synthetic images. ResNet18 was trained again with the same recipe and validation set as previously but now with 5000 training images instead of 4000. With 4000 training images and no synthetic data, an L1 loss on the validation set of 0.0156 ± 0.0007 and a MAPE of 18.934 ± 1.809% was achieved. In comparison, with 5000 training images, an L1 loss of 0.0161 ± 0.0003 and a MAPE of 19.181 ± 0.314% was achieved. These values are in the same ballpark, and adding the extra 1000 images only resulted in a smaller variation over the 5 training runs. Similarly, fine-tuning ResNet18_100k with 1000 extra images does not lead to any significant improvement. With 4000 real images, the fine-tuned ResNet18 model achieves an L1 validation loss of 0.00820 ± 0.00007 and a MAPE of 8.882 ± 0.136% over the 5 training runs, and with 5000 real images, the L1 loss is 0.00822 ± 0.00006 and the MAPE 8.873 ± 0.108%. From this, we conclude that generating a large and varied set of high quality synthetic data is significantly more resource efficient compared to generating more multislice images.

The performance of the size-estimator is ultimately judged by how well it is generalized to real experimental images. Thus, we apply ResNet18_100k_FT and U-Net_0 to 205 experimentally observed Pt particles. These particles were imaged with time-series, and several frames are available for all particles. We assume that the particle maintains its size throughout the time-series and the error of a size prediction in number of atoms is calculated as
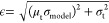
where μ_t_ and σ_t_ are the mean and standard deviation of the model predictions from all frames of a time-series. The model error in percent, σ_model_, depends on which size interval μ_t_ falls into, as specified in Figure 3c. As there is no ground truth size available for experimentally observed particles, judging whether predictions are reasonable is unavoidably biased and highly dependent on the opinion of domain experts. However, until smarter models are available that can physically explain why a certain size was predicted, alternatively predicting the entire 3D structure instead of just a size, manual verification is the best we can do. The authors’ opinions as domain experts are that ResNet18_100k_FT produces predictions that cannot be ruled out as unreasonable for about 80% of the 205 particles, while the same can only be said for about 70% of the U-Net_0 predictions, indicating that the wide variety of the synthetic data set is beneficial for model generalization to real-world data. For transparency, the predictions to all 205 experimental particles can be found as a collage in supporting Figure 1. A few selected predictions are displayed in Figure 4 where we also compare the network predictions to the common hemisphere approximation where the particle is modeled as a hemisphere with diameter manually measured from the image.

**Figure 4:**
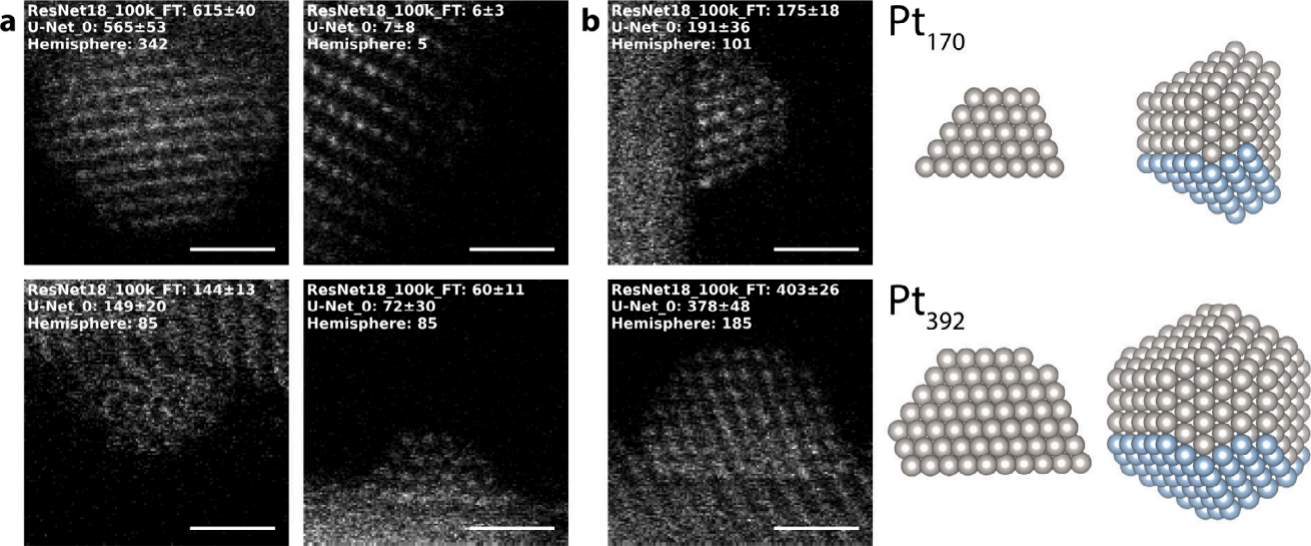
(a) Selected real experimental images with the corresponding ResNet18_100k_FT and U_Net_0 size predictions compared with the hemisphere estimate. Scale bars are 1 nm. (b) Selected real experimental images with corresponding predictions and matching atomic models. Atoms highlighted in blue are the columns visible in the side-view.

To further verify the validity and precision of predictions for some highly crystalline particles, we also construct example atomistic models with atom counts similar to those of the prediction. These models were designed to fit the projection of the particle in the image and are inspired by truncated octahedra, a commonly observed structure in Pt/CeO_2_.^30^ Two such models and their corresponding experimental images and size predictions are presented in Figure 4b. Although these models are constructed to align with the predictions, they are reasonable representations and are helpful for putting the predictions into perspective. The discrepancy between the model predictions and the hemisphere estimates also makes the limitations of that approximation clear.

In conclusion, a neural network based on a 3D U-Net was developed and trained to predict HAADF-STEM multislice images from atomic models. The network achieves a 100 times speedup over physical multislice simulations while maintaining high image quality, and it was used to generate 100000 images of Pt particles supported on CeO_2_. We find that generating a large synthetic data set is resource efficient compared to generating more real multislice images and that training on a large data set leads to significantly increased model performance. Out of the investigated architectures, ResNet18 performs the best and reduces the MdAPE of size predictions to 5.26% from 10.64% and the error standard deviation to 14.00% from 22.25% and increases the fraction of reasonable guesses on real experimental data to 80% from 70% compared to our previous work with a U-Net model trained without synthetic data. With these improvements, we believe that the technique is reliable and accurate and will be of great use for statistical STEM studies of heterogeneous catalysts to establish structure–property relationships. We predict that further advances of this methodology will come from working with a single pixel size (fixed magnification), automatic data acquisition,^31,32^ and less noisy experimental data and by generating more realistic structural models. Future studies could investigate the number of multislice images needed to train a good enough surrogate model and if faster alternatives to the multislice algorithm^14^ lead to similar prediction quality and generalization to experimental data.

## Supporting Information Available

Size predictions of ResNet18_100k_FT and U-Net_0 for all 205 experimentally observed Pt nanoparticles (PDF)

## References

[ref1] Mitchell S.; Pérez-Ramírez J. Atomically precise control in the design of low-nuclearity supported metal catalysts. Nature Reviews Materials 2021, 6 (11), 969–985. 10.1038/s41578-021-00360-6.

[ref2] Mitchell S.; Qin R.; Zheng N.; Pérez-Ramírez J. Nanoscale engineering of catalytic materials for sustainable technologies. Nat. Nanotechnol. 2021, 16 (2), 129–139. 10.1038/s41565-020-00799-8. 33230317

[ref3] Pyrz W. D.; Buttrey D. J. Particle Size Determination Using TEM: A Discussion of Image Acquisition and Analysis for the Novice Microscopist. Langmuir 2008, 24 (20), 11350–11360. 10.1021/la801367j. 18729338

[ref4] Mitchell S.; Parés F.; Faust Akl D.; Collins S. M.; Kepaptsoglou D. M.; Ramasse Q. M.; Garcia-Gasulla D.; Pérez-Ramírez J.; López N. Automated Image Analysis for Single-Atom Detection in Catalytic Materials by Transmission Electron Microscopy. J. Am. Chem. Soc. 2022, 144 (18), 8018–8029. 10.1021/jacs.1c12466. 35333043

[ref5] Rossi K.; Ruiz-Ferrando A.; Akl D. F.; Abalos V. G.; Heras-Domingo J.; Graux R.; Hai X.; Lu J.; Garcia-Gasulla D.; López N.; et al. Quantitative Description of Metal Center Organization and Interactions in Single-Atom Catalysts. Adv. Mater. 2024, 36 (5), 2307991 10.1002/adma.202307991. 37757786

[ref6] De Backer A.; van den Bos K. H. W.; Van den Broek W.; Sijbers J.; Van Aert S. StatSTEM: An efficient approach for accurate and precise model-based quantification of atomic resolution electron microscopy images. Ultramicroscopy 2016, 171, 104–116. 10.1016/j.ultramic.2016.08.018. 27657649

[ref7] De Backer A.; Martinez G. T.; Rosenauer A.; Van Aert S. Atom counting in HAADF STEM using a statistical model-based approach: Methodology, possibilities, and inherent limitations. Ultramicroscopy 2013, 134, 23–33. 10.1016/j.ultramic.2013.05.003. 23759467

[ref8] Eliasson H.; Lothian A.; Surin I.; Mitchell S.; Pérez-Ramírez J.; Erni R. Precise Size Determination of Supported Catalyst Nanoparticles via Generative AI and Scanning Transmission Electron Microscopy. Small Methods 2024, 2401108 10.1002/smtd.202401108. PMC11926506 39359026

[ref9] Zhu J. Y.; Park T.; Isola P.; Efros A. A. Unpaired Image-to-Image Translation Using Cycle-Consistent Adversarial Networks. In 2017 IEEE International Conference on Computer Vision (ICCV), 2017; pp 2242–2251.

[ref10] Erni R.; Heinrich H.; Kostorz G. Quantitative characterisation of chemical inhomogeneities in Al-Ag using high-resolution Z-contrast STEM. Ultramicroscopy 2003, 94 (2), 125–133. 10.1016/S0304-3991(02)00249-8. 12505761

[ref11] Uhrig J.; Schneider N.; Schneider L.; Franke U.; Brox T.; Geiger A. Sparsity Invariant CNNs. In 2017 International Conference on 3D Vision (3DV), 2017; pp 11–20.

[ref12] Krizhevsky A.; Sutskever I.; Hinton G. E. ImageNet classification with deep convolutional neural networks. In Proceedings of the 26th International Conference on Neural Information Processing Systems - Volume 1, Lake Tahoe, NV, 2012; pp 1097–1105.

[ref13] Rangel DaCosta L.; Brown H. G.; Pelz P. M.; Rakowski A.; Barber N.; O’Donovan P.; McBean P.; Jones L.; Ciston J.; Scott M. C.; et al. Prismatic 2.0 - Simulation software for scanning and high resolution transmission electron microscopy (STEM and HRTEM). Micron 2021, 151, 103141 10.1016/j.micron.2021.103141. 34560356

[ref14] Ophus C. A fast image simulation algorithm for scanning transmission electron microscopy. Adv. Struct. Chem. Imag. 2017, 3 (1), 13 10.1186/s40679-017-0046-1. PMC5423922 28546904

[ref15] Ros G.; Sellart L.; Materzynska J.; Vazquez D.; Lopez A. M. The SYNTHIA Dataset: A Large Collection of Synthetic Images for Semantic Segmentation of Urban Scenes. In 2016 IEEE Conference on Computer Vision and Pattern Recognition (CVPR), 2016; pp 3234–3243.

[ref16] Lin R.; Zhang R.; Wang C.; Yang X.-Q.; Xin H. L. TEMImageNet training library and AtomSegNet deep-learning models for high-precision atom segmentation, localization, denoising, and deblurring of atomic-resolution images. Sci. Rep. 2021, 11 (1), 5386 10.1038/s41598-021-84499-w. 33686158 PMC7940611

[ref17] Eliasson H.; Erni R. Localization and segmentation of atomic columns in supported nanoparticles for fast scanning transmission electron microscopy. Npj Comput. Mater. 2024, 10 (1), 168 10.1038/s41524-024-01360-0. 39104782 PMC11297796

[ref18] Richter S. R.; Vineet V.; Roth S.; Koltun V. Playing for data: Ground truth from computer games. In Computer Vision-ECCV 2016:14th European Conference, Amsterdam, The Netherlands, October 11–14, 2016, Proceedings, Part II, 2016; Springer: pp 102–118.

[ref19] LeBeau J. M.; Findlay S. D.; Allen L. J.; Stemmer S. Quantitative Atomic Resolution Scanning Transmission Electron Microscopy. Phys. Rev. Lett. 2008, 100 (20), 206101 10.1103/PhysRevLett.100.206101. 18518557

[ref20] Combs A. H.; Maldonis J. J.; Feng J.; Xu Z.; Voyles P. M.; Morgan D. Fast approximate STEM image simulations from a machine learning model. Adv. Struct. Chem. Imag. 2019, 5 (1), 2 10.1186/s40679-019-0064-2.

[ref21] He K.; Zhang X.; Ren S.; Sun J. Deep Residual Learning for Image Recognition. In 2016 IEEE Conference on Computer Vision and Pattern Recognition (CVPR), 2016; pp 770–778.

[ref22] Qi C. R.; Yi L.; Su H.; Guibas L. J. PointNet++: deep hierarchical feature learning on point sets in a metric space. In Proceedings of the 31st International Conference on Neural Information Processing Systems, Long Beach, California, USA, 2017; pp 5105–5114.

[ref23] Çiçek Ö.; Abdulkadir A.; Lienkamp S. S.; Brox T.; Ronneberger O. 3D U-Net: learning dense volumetric segmentation from sparse annotation. In Medical Image Computing and Computer-Assisted Intervention-MICCAI 2016:19th International Conference, Athens, Greece, October 17–21, 2016, Proceedings, Part II, 2016; Springer: pp 424–432.

[ref24] Multislice surrogate repository. https://github.com/benke97/MultisliceSurrogate (accessed 2025-01-18).

[ref25] Zhang R.; Isola P.; Efros A. A.; Shechtman E.; Wang O. The Unreasonable Effectiveness of Deep Features as a Perceptual Metric. In 2018 IEEE/CVF Conference on Computer Vision and Pattern Recognition (CVPR), 2018; pp 586–595.

[ref26] Iandola F. N.; Han S.; Moskewicz M. W.; Ashraf K.; Dally W. J.; Keutzer K. SqueezeNet: AlexNet-level accuracy with 50x fewer parameters and < 0.5MB model size. In Proceedings of the International Conference on Learning Representations (ICLR), 2017.

[ref27] Barthel J. Dr. Probe: A software for high-resolution STEM image simulation. Ultramicroscopy 2018, 193, 1–11. 10.1016/j.ultramic.2018.06.003. 29906518

[ref28] Pennycook S. J.; Jesson D. E. High-resolution Z-contrast imaging of crystals. Ultramicroscopy 1991, 37 (1), 14–38. 10.1016/0304-3991(91)90004-P.

[ref29] Kirkland E. J. Multislice Applications and Examples. In Advanced Computing in Electron Microscopy, Kirkland E. J., Ed.; Springer US, 2010; pp 163–197.

[ref30] Eliasson H.; Niu Y.; Palmer R. E.; Grönbeck H.; Erni R. Support-facet-dependent morphology of small Pt particles on ceria. Nanoscale 2023, 15 (47), 19091–19098. 10.1039/D3NR04701F. 37929917 PMC10701474

[ref31] Pattison A. J.; Pedroso C. C. S.; Cohen B. E.; Ondry J. C.; Alivisatos A. P.; Theis W.; Ercius P. Advanced techniques in automated high-resolution scanning transmission electron microscopy. Nanotechnology 2024, 35 (1), 015710 10.1088/1361-6528/acf938. 37703845

[ref32] Slater T.; Wang Y.-C.; McCormack J.; Leteba G.; Quiroz J.; Camargo P.; Palmer R.; Haigh S.; Allen C. Automating 3D Imaging of Inorganic Nanoparticles. Microsc. Microanal. 2021, 27 (S1), 2864–2866. 10.1017/S1431927621009995.

