# Supplementary material for: Improving Nanoparticle Size Estimation from Scanning Transmission Electron Micrographs with a Multislice Surrogate Model
Source: Nano Lett. 2025 Jan 29;25(6):2474–9. doi: 10.1021/acs.nanolett.4c06025 (PMC11827102; doi:10.1021/acs.nanolett.4c06025)
Supplement: Supplementary file 1 — nl4c06025_si_001.pdf [file nl4c06025_si_001.pdf]

Supporting Information

Improving Nanoparticle Size Estimation from Scanning Transmission Electron Micrographs with a Multislice Surrogate Model

Henrik Eliasson[a*] and Rolf Erni[a, b]

[a]*Electron Microscopy Center, Empa – Swiss Federal Laboratories for Materials Science and Technology, Überlandstrasse 129, 8600 Dübendorf, Switzerland.*

[b]*Department of Materials, ETH Zürich, CH-8093 Zürich, Switzerland.*

Email: henrik.eliasson@empa.ch

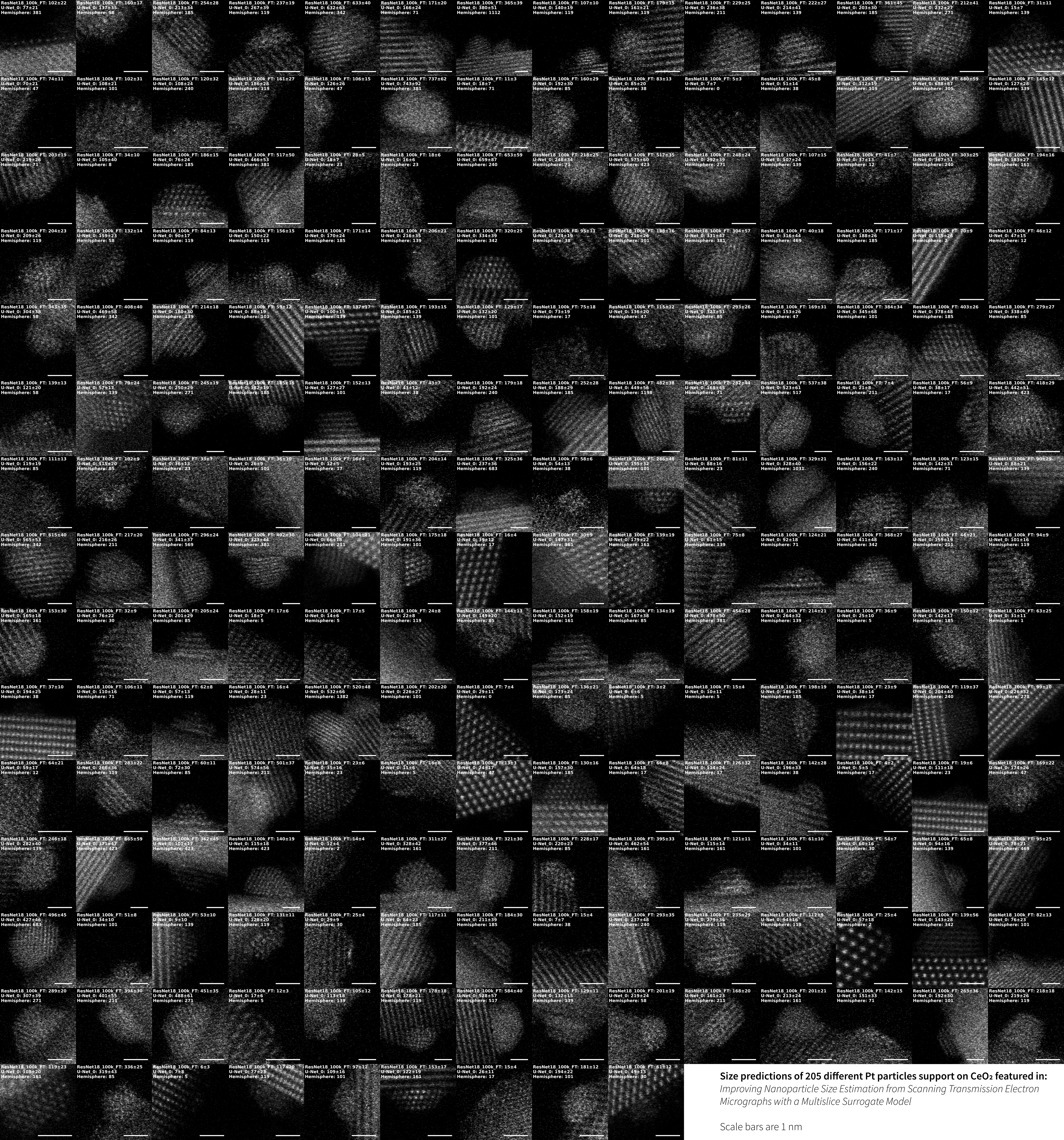

Size predictions of 205 different Pt particles support on CeO_2 featured in:
Improving Nanoparticle Size Estimation from Scanning Transmission Electron Micrographs with a Multislice Surrogate Model

Scale bars are 1 nm
